# Society Meetings

**Published:** 1903-05

**Authors:** 


### SOCIETY MEETINGS.

The National Association of United States Pension Examining Surgeons, will hold its second annual meeting at Washington, D.C., May 13-14, 1903. The secretary, Dr. Wheelock Rider, of Rochester, N. Y., has prepared the following program: Address of welcome, Hon. E. F. Ware, Commissioner of Pensions; Rheumatoid affections considered as pensionable disabilities, P. Y. Eisenberg, Norristown, Pa.; Physical signs of the heart: How detected and how described, C. W. S. Frost, Waterbury, Conn.; The etiology of some pensioned disabilities measured by modern pathology, W. T. Sarles, Sparta, Wis.; Diseases of the respiratory tract, William Warren Potter, Buffalo, N.Y.; Address, Sam Houston, Medical Referee, Bureau of Pensions, Washington (by invitation); Insanity in connection with the service in the Philippine Islands, Ernest F. Robinson, surgeon U. S. navy, Kansas City, Mo. (by invitation); A cursory survey of the pension examiner's work, G. Law, Greeley, Col.; Hints to the general board surgeon on eye and ear examinations, J. O. Stillson, Indianapolis, Ind.; Diagnosis of malaria and its sequelæ, John C. Hemmeter, director, clinical laboratory, Baltimore, Md., University of Maryland (by invitation); Insanity as a result of physical conditions, A. B. Richardson, superintendent Government Hospital for the Insane, Washington (by invitation); President's address, William A. Howe, Phelps, N. Y.

The headquarters of the association will be at the Riggs

House, 15th and G. Streets, and the place of meeting will be at hall of Georgetown Law School, E Street, between 5th and 6th Streets.

On invitation of the Commissioner of Pensions, the members will visit the Pension Bureau, Wednesday or Thursday afternoon. A reception for members and their families has been arranged for Wednesday evening.

On convening at 10 a.m. on Wednesday the association will consider the report of the executive committee, the proposed constitution and by-laws, reports of secretary and of treasurer, etc. The final business session will be held on Thursday afternoon.

---

THE Buffalo Academy of Medicine held meetings during the months of March and April, 1903, as follows:

*Section on Ophthalmology, Otology and Laryngology.*—Stated meeting, Tuesday, March 31. Program: A consideration of the value of topical applications to the upper air tract, F. Whitehill Hinkel.

*Section on Surgery.*—Tuesday evening, April 7. Program: The rôle of cholecystitis in acute inflammation of the peritoneum, with report of cases, W. G. Macdonald, Albany, N. Y.

Tuesday evening, April 14. Program: The reduction of the eye in cave animals, (illustrated by stereopticon), Prof. Carl Eigenmann, Indiana University, Bloomington, Ind.

*Section on Obstetrics and Gynecology.*—Tuesday evening, April 21. Program: Injuries to the parturient canal during labor; their prevention and treatment, C. E. Congdon. Complications of gonorrhea in the female, H. D. Ingraham.

---

THE American Medical Association will hold its 54th annual meeting at New Orleans, May 5-8, 1903. The Illinois Central Railroad (see adv. p. xxxi.) offers splendid accommodations, via Cincinnati or Chicago, at one fare for the round trip. For particulars G. B. Wyllie, General Passenger Agent, 210 Ellicott Square, Buffalo, should be consulted.

---

A SANITARY CONGRESS which will consider matters of international importance will be held at Bradford, England, from July

7 to 11, 1903. Ambassador Choate has informed the state department at Washington of this fact, and that the United States is invited to send representatives to the congress.

---

The American Academy of Medicine will hold its 28th annual meeting at the Arlington Hotel, Washington, D. C., Monday and Tuesday, May 11 and 12, 1903, under the presidency of Dr. Charles McIntire, of Easton, Pa.

